# Effects of Chronic Administration of Capsaicin on Biomarkers of Kidney Injury in Male Wistar Rats with Experimental Diabetes

**DOI:** 10.3390/molecules24010036

**Published:** 2018-12-21

**Authors:** Mónica Ríos-Silva, Rubén Santos-Álvarez, Xóchitl Trujillo, Rosa Yolitzy Cárdenas-María, Marisa López-Zamudio, Jaime Alberto Bricio-Barrios, Caridad Leal, Alfredo Saavedra-Molina, Miguel Huerta-Trujillo, Karina Espinoza-Mejía, Miguel Huerta

**Affiliations:** 1 Universidad de Colima, Centro Universitario de Investigaciones Biomédicas, Av. 25 de Julio No. 965, Col. Villas San Sebastián, Colima 28045, Colima, Mexico; mrios@ucol.mx (M.R.-S.); rsantos2@ucol.mx (R.S.-A.); rosio@ucol.mx (X.T.); rosa_cardenas@ucol.mx (R.Y.C.-M.); mlopez21@ucol.mx (M.L.-Z.); xtrujillo@ucol.mx (K.E.-M.); 2 Universidad de Colima-Cátedras-CONACyT, Centro Universitario de Investigaciones Biomédicas, Av. 25 de Julio No. 965, Col. Villas San Sebastián, Colima 28045, Colima, Mexico; 3 Universidad de Colima, Facultad de Medicina, Escuela de Nutrición, Av. Universidad No. 333, Col. Las Víboras, Colima 28040, Colima, Mexico; jbricio@ucol.mx; 4 Instituto Mexicano del Seguro Social, Centro de Investigaciones Biomédicas de Occidente, Sierra Mojada No. 800, Col. Independencia, Guadalajara 44340, Jalisco, Mexico; lealc36@yahoo.com.mx; 5 Universidad Michoacana de San Nicolás de Hidalgo, Instituto de Investigaciones Químico-Biológicas; Av. Francisco J Mujica S/N, Morelia 58020, Michoacán, Mexico; saavedra@umich.mx; 6 Universidad de Colima, Facultad de Medicina, Av. Universidad No. 333, Col. Las Víboras, Colima 28040, Colima, Mexico; miguel.htrujillo@gmail.com

**Keywords:** TRPV1, capsaicin, renal injury, diabetes mellitus

## Abstract

Capsaicin is an agonist of the transient receptor potential vanilloid type 1 (TRPV1) channel, which has been related to the pathophysiology of kidney disease secondary to diabetes. This study aimed to evaluate the chronic effect of capsaicin administration on biomarkers of kidney injury in an experimental rat model of diabetes. Male Wistar rats were assigned to four groups: (1) healthy controls without diabetes (CON), (2) healthy controls plus capsaicin at 1 mg/kg/day (CON + CAPS), (3) experimental diabetes without capsaicin (DM), and (4) experimental diabetes plus capsaicin at 1 mg/kg/day (DM + CAPS). For each group, 24-h urine samples were collected to determine diuresis, albumin, cystatin C, β2 microglobulin, epidermal growth factor (EGF), alpha (1)-acid glycoprotein, and neutrophil gelatinase-associated lipocalin (NAG-L). Blood samples were drawn to measure fasting glucose. After 8 weeks, the CON + CAPS and DM + CAPS groups showed increased diuresis compared to the CON and DM groups, but the difference was significant only in the DM + CAPS group. The two-way ANOVA only showed a statistically significant effect of CAPS on the urinary EGF levels, as well as a tendency to have a significant effect in the urinary NAG-L levels. The EGF levels decreased in both CAPS-treated groups, but the change was only significant in the CON + CAPS group vs. CON group; and the NAG-L levels were lower in both CAPS-treated groups. These results show that capsaicin had a diuretic effect in healthy and diabetic rats; additionally, it increased the urinary EGF levels and tended to decrease the urinary NAG-L levels.

## 1. Introduction

Diabetic nephropathy (DN) is a clinical syndrome characterized by persistent albuminuria, elevated blood pressure, progressive reduction of the glomerular filtration rate (GFR), and increased risk of cardiovascular morbidity and mortality [1]. The natural evolution of DN, without treatment, leads to chronic kidney failure (CKF). To prevent disease progression, the preferred treatment is antihypertensive drugs, such as angiotensin-converting enzyme blockers and angiotensin II receptor blockers, which reduce the progression of kidney injury [2,3].

The transient receptor potential vanilloid type 1 (TRPV1) is a six transmembrane domain nociceptive transducer that forms a non-selective cationic channel with an affinity for calcium. This channel has been extensively described in sensory nerve fibers, but it has also been described in other organs, including the kidney [4]. TRPV1 was reported to be involved in hemodynamic and electrolyte homeostasis. Its activation reduced renal perfusion pressure and increased both the GFR and renal excretion by increasing natriuresis and diuresis [5,6]. Capsaicin (CAPS), a natural alkaloid extracted from chili pepper, is a selective agonist of TRPV1. In animal models of renal toxicity (e.g., with cis-platinum treatment), the administration of CAPS reduced kidney injury. CAPS treatment also reduced the expression of inflammation markers and oxidative stress [7]. However, it was recently reported that CAPS could also activate cannabinoid brain type 1 (CB1) receptors [8]. CB1 receptors were reported to be expressed in podocytes [9]. Additionally, it was shown that in DN, the CB1 receptor expression was elevated, and type 2 cannabinoid receptor (CB2) expression was diminished. The interaction of the endocannabinoid system with the renin-angiotensin-aldosterone system provided evidence that CB1 inhibition reduced the expression of angiotensin type 1 receptor (AT1R). Thus, the effect of blocking CB1 might be similar to an AT1R blockade [10,11,12].

Activation of TRPV1 by CAPS was shown to affect kidney physiological and pathophysiological processes, and it sometimes had nephroprotective effects. However, to our knowledge, it is the first report to describe the effect of chronic CAPS administration on early biomarkers of renal injury in a diabetes model.

This study aimed to measure the effects of chronic subcutaneous administration (8 weeks) of CAPS (1 mg/kg/day) on early biomarkers of renal injury under conditions of diabetic hyperglycemia and normoglycemia in male Wistar rats.

## 2. Results and Discussion

Kidney disease secondary to diabetes represents the main cause of long-term, chronic, end-stage kidney disease [1]. When poor kidney function is detected with classical indicators, like urea and creatinine, it implies that the pathophysiological process of kidney disease is in the advanced stages. Therefore, it is desirable to identify other markers, which can be detected before changes in urea and creatinine appear, to indicate the earlier stages of kidney failure [13]. In the present study, we evaluated the molecules that are eliminated by the kidney, which have been shown to play a role in the physiopathology of this chronic disease, secondary to diabetes. These biomarkers reflect glomeruli/podocyte status and tubular damage. In addition, they were postulated to be useful in identifying the earlier steps of diabetic nephropathy.

At the beginning of this study, we found no differences in glycemia or body weight between the two groups of healthy and the two groups of diabetic rats (Table 1). Similarly, although the final values of glucose and body weight were different between the diabetic and healthy groups, they were not different between the groups treated with and without CAPS.

### 2.1. Diuretic Effect of Capsaicin in Diabetic and Healthy Rats

**Figure 1:**
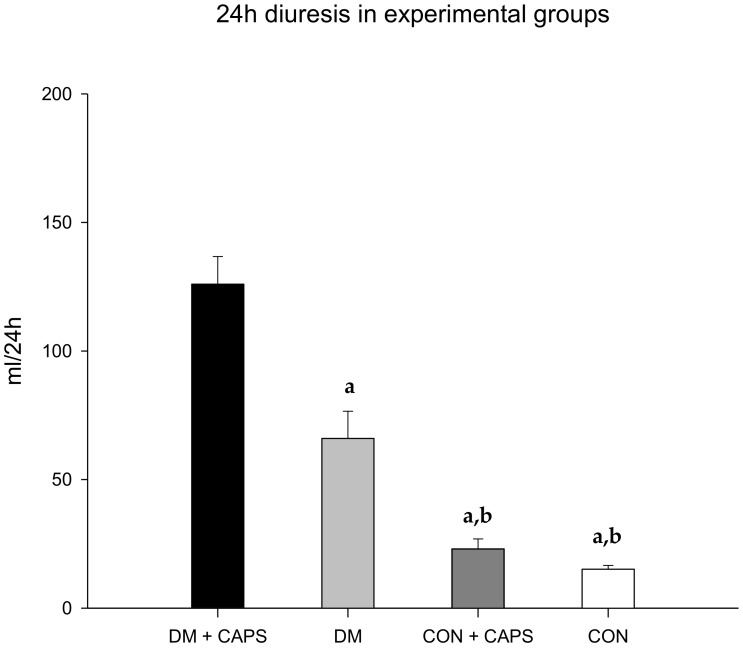
Effect of 8 weeks of capsaicin administration (1 mg/kg/day) on diuresis. (**a**) Statistically significant difference in comparison with the DM group + CAPS; (**b**) statistically significant difference in comparison with the DM group. Data are mean ± SEM.

After 8 weeks of treatment, all rats treated with CAPS exhibited increased diuresis, but the effect was only significant in diabetic rats. (Figure 1). This result indicated that there was an interaction between CAPS treatment and diabetes, which had an effect on diuresis (Table 2).

It is known that TRPV1 is expressed in kidney tissues, including the parenchyma, nerves, and arteries [14,15,16]. At the renal pelvis, sensory nerve endings with positive TRPV1 expression participate in the reno-renal reflex. This reflex is triggered by an increment in the pressure, which is sensed by mechanoreceptors, and changes in salt concentrations, which are sensed by chemoreceptors. These stimulations cause changes in renal afferent and efferent nerve signaling, which control urinary excretion and flow. CAPS administration increased the activity of the renal afferents responsible for this reflex, which resulted in increased natriuresis and diuresis [6,16]. These increases in diuresis and natriuresis have been associated with renal vasodilatation. Thus, dilatation of afferent arterioles could lead to increases in GFR and diuresis-natriuresis, as observed in some animal models [17]. Interestingly, this mechanosensory response was diminished in STZ-induced diabetic rats [18], and improvements in glycemic control could improve the signaling involved in this response.

Here, we observed that in groups with similar levels of glycemia, CAPS increased diuresis. These findings suggested that the afferent and efferent pathways were conserved in diabetic rats treated with CAPS. Alternatively, CAPS might have acted through another pathway that did not involve TRPV1 receptors, either directly or indirectly. For example, CAPS might have acted through CB1 receptors, whose activation has also been associated with increased diuresis [19]. More research is needed to determine whether the diuretic effect of CAPS can confer benefits in diabetes mellitus complicated with other comorbidities, such as hypertension, cardiac failure, or advanced stages of kidney disease [20,21].

### 2.2. Effect of CAPS on Biomarkers of Kidney Injury

Several studies have reported that TRPV1 agonists, like CAPS, could protect against kidney injury in several different pathologies, such as hypertension, acute renal injury (due to ischemia-reperfusion), and toxicity from cisplatin chemotherapy. However, no study has demonstrated a protective effect of CAPS in kidney disease due to DN [22]. Accordingly, to our knowledge, this was the first study to explore the chronic effects of CAPS on early indicators of renal injury in a diabetic model. We found that, as expected, most kidney injury indicators in the DM groups were elevated (except epidermal growth factor (EGF), which was reduced because it is negatively correlated with kidney injury) compared to the healthy rat groups. The unexpected result was that alpha (1) acid glycoprotein (AGP) levels were similar across all groups (Table 2) [23].

**Figure 2:**
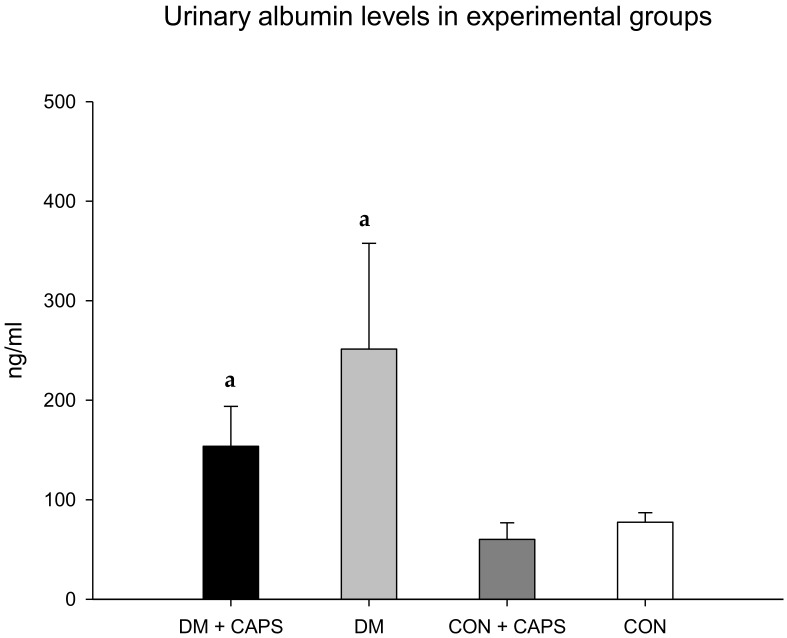
Effect of 8 weeks of capsaicin administration (1 mg/kg/day) on urinary albumin levels. (**a**) Statistically significant difference in comparison with the CON healthy group + CAPS. Data are mean ± SEM.

Figure 2 shows that urinary albumin levels tended to be lower in rats treated with CAPS than in untreated rats; however, the differences were not statistically significant. Albuminuria is the earliest detectable and most widely used biomarker of kidney disease. Urinary albumin excretion is associated with damaged glomeruli. Generally, small quantities of albumin are filtered through the three glomerular layers: the endothelial fenestrae, the basal glomerular membrane, and podocytes. Most of this filtered albumin is reabsorbed in the convoluted proximal tubule; however, any increment in albumin filtration could saturate tubular transport mechanisms and result in proteinuria. Chronic hyperglycemia is the main stimulus for inducing damage to the three glomeruli layers. Consequently, urinary albumin is often used as prognostic biomarker for evaluating the risk of kidney disease secondary to diabetes [24]. It has been shown that albuminuria was increased in an animal model that developed hypertension due to a TRPV1 knockout. Conversely, in AKD models or in cisplatin-treated animals, albuminuria decreased after treatment with CAPS [5,7,25].

**Figure 3:**
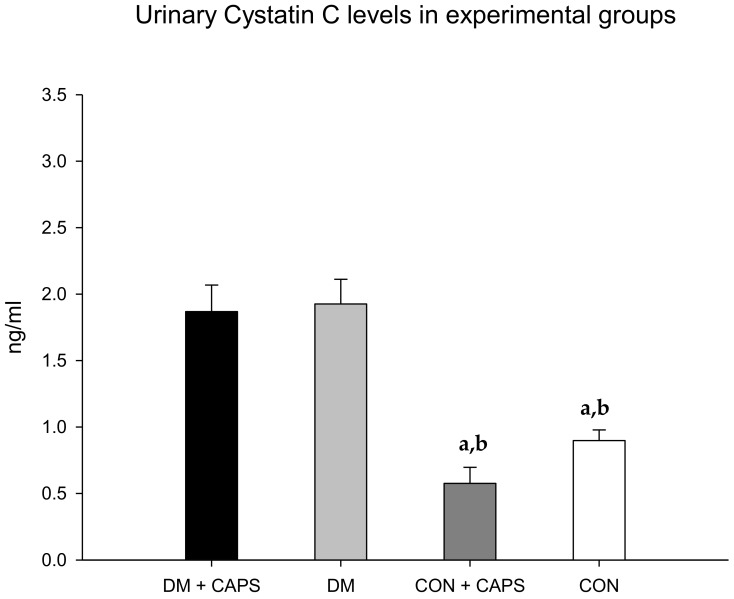
Effect of 8 weeks of capsaicin administration (1 mg/kg/day) on urinary cystatin C levels. (**a**) Statistically significant difference in comparison with the DM group + CAPS; (**b**) statistically significant difference in comparison with the DM group. Data are 3 mean ± SEM.

The levels of cystatin C were lower with CAPS treatment, but only between untreated and CAPS-treated healthy rats; moreover, the difference was not statistically significant (Figure 3). Cystatin C is a low-molecular-weight cysteine protease inhibitor protein, derived from nucleated cells. Like albumin, once cystatin C is filtered, it is mostly reabsorbed in the proximal tubule. In the presence of proximal tubule damage, its levels are increased in the urine. Previous studies showed that cystatin C levels were increased in diabetic rats [26].

**Figure 4:**
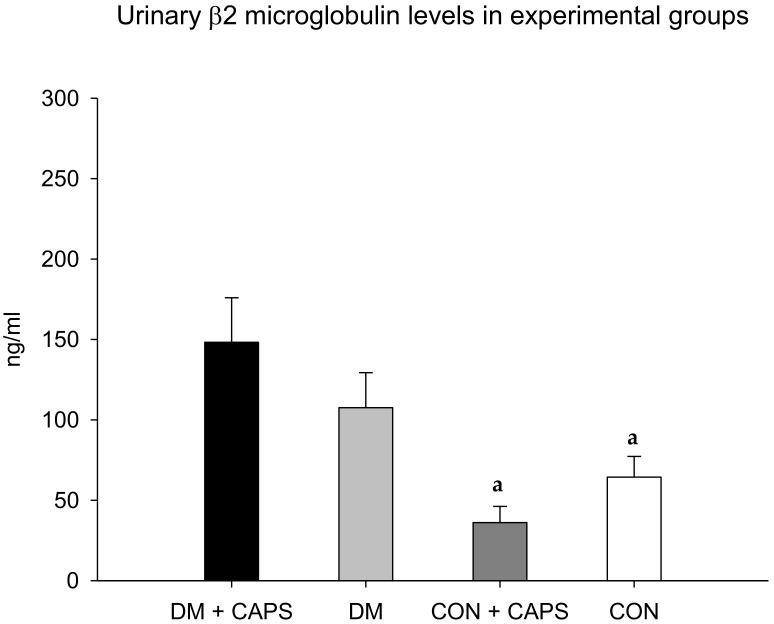
Effect of 8 weeks of capsaicin administration (1 mg/kg/day) on urinary β2 microglobulin levels. (**a**) Statistically significant difference in comparison with the DM group + CAPS. Data are mean ± SEM.

On the other hand, the β2M levels in diabetic rats treated with CAPS were significantly higher than the levels in both healthy rat groups, but not significantly different from the level in the diabetic control group (Figure 4). β2M is a low-molecular-weight protein that participates in the immune response. β2M is essential for antigen presentation by the major histocompatibility complex. β2M is also involved in albumin and iron homeostasis. Most (99%) of the filtered β2M is reabsorbed in the convoluted proximal tubule by a mechanism mediated by the megalin-cubilin complex. It is then metabolized by tubular cells. Thus, high urinary β2M levels are considered to be a biomarker of tubular damage [27].

We could not find any previous reports that measured the effect of CAPS or TRPV1 agonists on urinary cystatin C or β2M.

**Figure 5:**
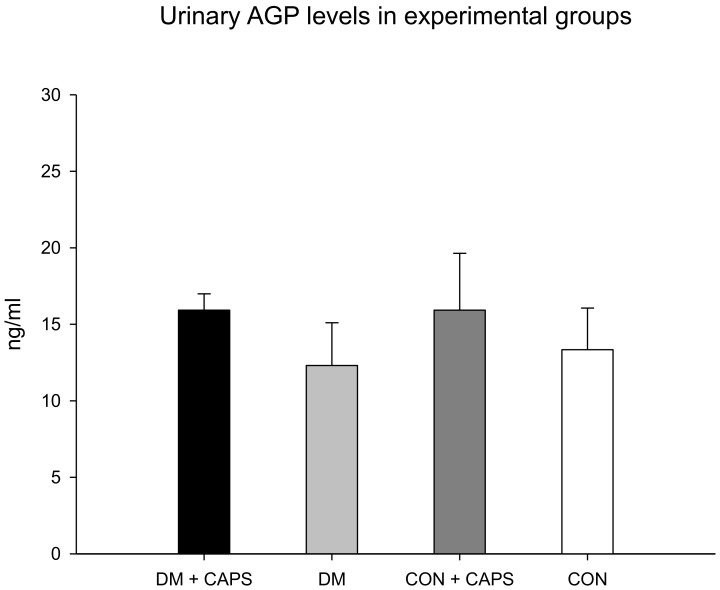
Effect of 8 weeks of capsaicin administration (1 mg/kg/day) on urinary AGP levels. AGP: alpha (1)-acid glycoprotein. Data are mean ± SEM.

AGP is a glycoprotein produced by the liver; it is considered an acute-phase reactant. It acts in glomeruli after activation by cytokines, including interleukin-1 beta and TNF-alpha. AGP has been referred to as an indicator of active kidney disease in inflammatory pathologies [28,29]. AGP levels were not different between untreated and CAPS-treated rats or between healthy and diabetic groups (Figure 5).

**Figure 6:**
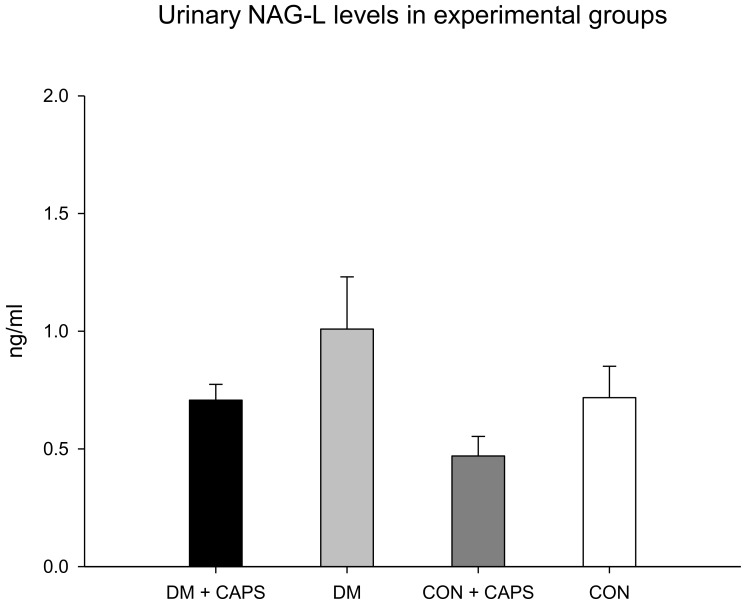
Effect of 8 weeks of capsaicin administration (1 mg/kg/day) on urinary NAG-L levels. NAG-L: Neutrophil-gelatinase associated lipocalin. Data are mean ± SEM.

Neutrophil gelatinase-associated lipocalin (NAG-L) levels were only significantly different between untreated diabetic rats and healthy rats treated with CAPS. However, in our study groups, the NAG-L levels were not significantly affected by either diabetes alone or by an interaction between diabetes and CAPS treatment (Figure 6). NAG-L is a glycoprotein that belongs to a family of small hydrophobic ligand transporters with antioxidant functions. In addition, NAG-L contributes to defending against bacterial infections. It is reabsorbed in the tubule through the same mechanism that functions to reabsorb albumin and β2M, but most NAG-L excretion is dependent on its secretion by the distal nephron. Thus, its presence in urine has been identified as a biomarker of damage at the level of the distal tubule [30]. It has been shown that elevated NAG-L in the urine is an indicator of kidney disease progression in individuals with diabetes [31]. In our experiment, the effect of CAPS treatment on the levels of this biomarker was not statistically significant, but the *p*-value was very close to significant (*p* = 0.051, Table 2). Figure 6 shows that NAG-L levels tended to decrease with CAPS treatment, in both diabetic and healthy rats.

**Figure 7:**
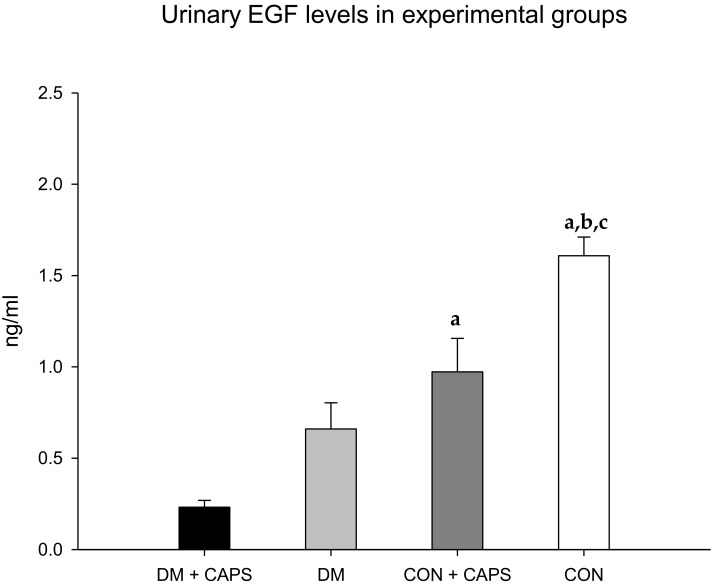
Effect of 8 weeks of capsaicin administration (1 mg/kg/day) on urinary EGF levels. (**a**) Statistically significant difference in comparison with the DM group + CAPS; (**b**) statistically significant difference in comparison with the DM group; (**c**) statistically significant difference in comparison with the CON group + CAPS. EGF: epidermal growth factor. Data are mean ± SEM.

Finally, EGF levels were reduced in both groups (healthy and diabetic rats) treated with CAPS, compared to their corresponding controls, but the reduction was statistically significant only in the healthy group (Figure 7). EGF has been identified as an indicator of both acute and chronic kidney failure. EGF appears to participate in the maintenance of tubular integrity [32]. Accordingly, acute and chronic kidney failure were associated with diminished levels of urinary EGF [33]. In contrast to other biomarkers, EGF excretion appears to depend on its production by juxtaglomerular cells in the kidney, rather than the filtration or reabsorption of plasma EGF [34]. In our study, CAPS significantly reduced the levels of urinary EGF in both healthy and diabetic rats. It is probable that, due to the chronic exposure imposed in the present study, CAPS might have acted directly on EGF production, and not through kidney filtration or reabsorption modifications. Previously, CAPS was reported to affect EGF in neoplastic cells. In those cells, EGF signaling decreased with CAPS treatment, and that effect was thought to contribute to the antitumoral activity of CAPS [35]. However, in kidney tissue, this reduction might increase the kidney’s susceptibility to injury. Indeed, in models of organ transplantation, EGF seemed to exert a tubular nephroprotective effect [33]. 

### 2.3. Study Limitations and Future Directions

It is known that TRPV1 receptors are differentially expressed according to sex in some tissues. Consequently, female rats might exhibit different effects from those observed in the male rats of the present study [36]. Additionally, the sexes have shown different predispositions to developing kidney injury due to diabetes [37]. Moreover, as mentioned above, previous studies have provided evidence that CAPS was capable of stimulating receptors other than TRPV1, like CB1 receptors [8], which were reported to be overexpressed in DN kidneys. Therefore, in future studies, we plan to use both sexes of rats when examining the CAPS effect together with an antagonist of the CB1 receptor.

## 3. Materials and Methods

### 3.1. Animals

Thirty-six male, 2-month-old, Wistar rats (Envigo, Inc. Huntingdon, UK) that weighed 250 ± 50 g were housed under pathogen-free conditions. All animals were maintained according to specific protocols for Laboratory Animals at facility of the University Center for Biomedical Research at the Universidad de Colima, México. Animals were housed in standard photoperiod conditions (12 h light/12 h dark), at 22 ± 2 °C ambient temperature. During the experiment, food and water were supplied ad libitum, except during food deprivation periods before blood draws.

All experimental protocols and animal management protocols were in accordance with the ethical standards and technical specifications of the Mexican Official Norm technical specifications for the production, care, and use of laboratory animals (NOM-062-ZOO-1999) and according to the recommendations for the care and use of laboratory animals from the National Institutes of Health.

### 3.2. Experimental Protocol

Animals were divided into four experimental groups: (1) a control group (healthy rats) that received no treatment (n = 8); (2) a control group that received CAPS (1 mg/kg/day) for eight weeks (n = 8), (3) a diabetic group that received no treatment (n = 10); and (4) a diabetic group that received CAPS treatment (1 mg/kg/day) for eight weeks, starting one week after the induction of diabetes (n = 10).

#### 3.2.1. Experimental Induction of Diabetes

To induce diabetes, rats received a single intraperitoneal administration of 45 mg/kg of streptozotocin (STZ, Sigma-Aldrich CO. St. Louis, MO, USA) [38] The diabetic state was confirmed with a fasting blood glucose measurement of ≥200 mg/dL [39].

#### 3.2.2. CAPS Dose and Administration

First, we prepared a vehicle solution with 10% Tween 80 (Sigma-Aldrich, St. Louis, MO, USA), 10% ethanol and 0.9% saline solution at a ratio of 2:1.1; then CAPS (Sigma-Aldrich, St. Louis, MO, USA) was dissolved in the vehicle in a final concentration of 3 mg/mL. This solution was administered subcutaneously immediately after preparation, with a CAPS dose of 1 mg/kg/day, in an injection volume of approximately 0.1 mL [39,40,41], for eight weeks.

#### 3.2.3. Fasting Glucose Measurement

Blood glucose was measured after 12 h of fasting. Briefly, peripheral blood was drawn from the tip of the tail, and glucose was measured with an Accu-Check Active auto analyzer (Roche, Mannheim, Germany).

#### 3.2.4. Determination of Kidney Injury Biomarkers

Animals were maintained in metabolic cages for 24-h urine collection. After 24 h, the collected urine was centrifuged at 400× *g* for 5 min, then it was frozen at −80 °C, until analysis. For analyses, thawed samples were divided into aliquots, diluted 1:500, and placed into 96-well plates. The plates contained magnetic pearls of a Toxicity Multiplex Assay kit (MILLIPLEX MAP Rat Kidney Toxicity Magnetic Bead Panel 2; Cat. RKTX2MAG-37K from EMD Millipore Corp., Charles, MO, USA). Analyses were performed according to the manufacturer´s recommendations to determine the levels of albumin, cystatin C, β2M, NGAL, EGF, and AGP. All biomarker concentrations were evaluated with a Magpix instrument from Luminex X-MAP.

### 3.3. Method of Euthanasia

Rats were euthanized without pain or distress with increasing anesthesia doses (intraperitoneal lethal doses with Pentobarbital Sodium, Pets pharm Mexico). The Ethics Committee from the Universidad de Colima approved all protocols (2017–08).

### 3.4. Statistical Analysis

We performed descriptive statistical analyses. The variables are expressed as the mean ± standard error of the mean. *P*-values <0.05 were considered statistically significant. Analyses were performed with IBM SPSS Statistics, version 22 (IBM Corp, Armonk, NY, USA). We first performed the two-way ANOVA test, then the Tukey post hoc analysis.

## 4. Conclusions

We evaluated the effect of chronic CAPS administration on indicators of renal injury in diabetic rats. We evaluated indicators of both glomerular and interstitial tubular injury, and each represented a different pathophysiological aspect of kidney disease secondary to diabetes. We observed that CAPS had a diuretic effect in this rat model of diabetes, but the effects of CAPS on indicators of kidney injury varied, depending on the indicator analyzed.

In both healthy and diabetic rats, CAPS had a diuretic effect, and additionally, it increased urinary EGF levels, and tended to decrease urinary NAG-L levels. More studies are needed to establish the role of TRPV1 and its signaling in the development and progression of kidney disease due to diabetes.

## Figures and Tables

**Table 1:** Mean fasting glucose levels and body weights of experimental groups at the beginning and end of the experiment.

Measurements	DM + CAPS	DM	CON + CAPS	CON	p^A^ for DM Effect	p^A^ for CAPS Effect	p^A^ for Interaction DM × CAPS Effect
Fasting Glucose (mg/dL) Initial	288.1 ± 24.7	326.0 ± 35.0	88.2 ± 3.4	81.5 ± 3.0	<0.001	0.4	0.3
Final	280.6 ± 29.4	294.5 ± 43.4	73.5 ± 3.0	74.6 ± 2.5	<0.001	0.7	0.8
Weight (g) Initial	280.4 ± 10.5	297.2 ± 4.2	291.1 ± 5.7	298.6 ± 3.6	0.4	0.1	0.05
Final	287.5 ± 10.6	306.0 ± 11.5	375.6 ± 10.7	349.6 ± 4.5	<0.001	0.7	0.03

CON: Control, DM: diabetes mellitus, CAPS: Capsaicin. Data are the mean ± SEM. ^A^ Two-way ANOVA.

**Table 2:** Two-way ANOVA analysis of the effects of DM, CAPS and their interaction on parameters related to early stages of kidney dysfunction.

Urinary Parameter	Source of Variance	SS	% variance	F	*p*
Uresis	DM effect	49847.4	73.9	84.8	<0.001
	CAPS effect	9698.9	35.5	16.5	<0.001
	DM × CAPS effect interaction	5720.0	24.5	9.7	0.004
Albumin	DM effect	150656.9	15.9	5.7	0.024
	CAPS effect	27813.3	3.4	1.0	0.315
	DM × CAPS interaction effect	13582.4	1.7	0.5	0.480
Cystatin C	DM effect	11.3	63.1	51.2	<0.001
	CAPS effect	0.3	4.3	1.3	0.255
	DM × CAPS interaction effect	0.1	2.1	0.6	0.423
β2 microglobulin	DM effect	50744.9	31.5	13.8	0.001
	CAPS effect	323.1	0.3	0.1	0.769
	DM × CAPS interaction effect	10017.8	8.3	2.7	0.109
AGP	DM effect	2.2	1	0.0	0.844
	CAPS effect	80.6	4.5	1.3	0.246
	DM × CAPS interaction effect	2.2	1	0.0	0.844
NAG-L	DM effect	0.6	11.5	3.8	0.058
	CAPS effect	0.6	12.2	4.1	0.051
	DM × CAPS interaction effect	0.0	0	0.0	0.836
EGF	DM effect	6.0	61.5	47.8	<0.001
	CAPS effect	2.3	38.7	18.9	<0.001
	DM × CAPS interaction effect	8.0	0.02	0.7	0.406

AGP: alpha (1)-acid glycoprotein, NAG-L: N-acetyl-b-D-glycosaminidase, EGF: epidermal growth factor. DM: Diabetes mellitus, CAPS: Capsaicin. SS: Sum of squares. Df = 1 for all cases.
